# Efficacy and Safety of a Novel Herbal Medicine in the Treatment of Irritable Bowel Syndrome: A Randomized Double-Blinded Clinical Trial

**DOI:** 10.1155/2020/8213082

**Published:** 2020-05-27

**Authors:** Ghasem Bordbar, Mohammad Bagher Miri, Mahmoud Omidi, Saeed Shoja, Malihe Akhavan

**Affiliations:** ^1^Student Research Committee, Hormozgan University of Medical Sciences, Bandar Abbas, Iran; ^2^Department of Gastroenterology, Shahid Mohammadi Hospital, Hormozgan University of Medical Sciences, Bandar Abbas, Iran; ^3^Department of Pharmacology and Toxicology, Faculty of Pharmacy and Pharmaceutical Sciences, Fertility and Infertility Research Center, Hormozgan University of Medical Sciences, Bandar Abbas, Iran; ^4^Infectious and Tropical Diseases Research Center, Hormozgan Health Institute, Hormozgan University of Medical Sciences, Bandar Abbas, Iran; ^5^Pharmaceutical Sciences Research Center, Department of Medicinal Chemistry, Mazandaran University of Medical Sciences, Sari, Iran

## Abstract

**Background:**

The unresponsiveness to conventional pharmacological treatments and their side effects have led patients with irritable bowel syndrome (IBS) to use complementary and alternative medicine such as herbal remedies. Beside, *Zataria multiflora* Boiss (ZM), *Trachyspermum ammi* L. (TA), and *Anethum graveolens* L. (AG) are being used as an antiseptic, carminative, and antispasmodic in traditional medicine. This trial investigated the efficacy and safety of a combination of ZM, AG, and TA essential oils in the treatment of IBS.

**Method:**

The present study was a randomized double-blind clinical trial with parallel groups in Iran. Patients in the control arm received three tablets of 10 mg hyoscine butylbromide daily for two weeks, and the intervention arm was daily treated with two 250 mg softgel capsules containing 180 mg of essential oils of ZM, AG, and TA for two weeks. Primary outcomes were the response rates based on the IBS Symptom Severity Scale (IBS-SSS), IBS Adequate Relief (IBS-AR), and IBS Global Assessment Improvement (IBS-GAI) at the end and two weeks after the end of the intervention. Secondary outcomes were the improvement rates in IBS-SSS scores, improving the quality of life, safety, and tolerability.

**Results:**

The posttreatment improvement percentage based on IBS-AR, IBS-GAI, and IBS-SSS scales was 83.9%, 75%, and 87% in the intervention group and 37.9%, 27.5%, and 34.4% in the control group, respectively (*P* < 0.001). Also, the improvement of the quality of life in the herbal medicine arm was significantly more than that in the control arm (*P* < 0.001).

**Conclusions:**

According to the results, the herbal medicine investigated in this study can be considered an appropriate alternative treatment for IBS.

## 1. Introduction

Irritable bowel syndrome (IBS) is a gastrointestinal syndrome characterized by chronic abdominal pain and a change in intestinal habits without any organic cause [1]. Since IBS include a variety of manifestations, its treatment depends on dominant symptoms and IBS type [2]. Compounds such as psyllium are prescribed to treat constipation-predominant IBS (IBS-C), and medicines such as loperamide are usually used for diarrhea-predominant IBS (IBS-D) [3, 4]. However, these medications do not improve all symptoms of IBS and exacerbate some symptoms such as bloating and abdominal crampy pains [5, 6]. Antispasmodic and anticholinergic medications are usually used to treat abdominal crampy pains in IBS patients, although they can worsen the symptoms in IBS-C patients [4, 7].

On the other hand, in IBS with mixed symptoms (IBS-M), taking these medications improves one type of symptoms but exacerbates the other type [4, 8, 9]. Various manifestations of IBS, improper response to conventional medicinal treatments and their adverse effects, and symptom recurrence have attracted the attention of patients towards complementary and alternative medicine (CAM) such as herbal medicines [10, 11]. In a way, almost 50% of IBS patients tend to take CAM [12]. *Zataria multiflora* Boiss (ZM), *Trachyspermum ammi* L. (TA), and *Anethum graveolens* L. (AG) are among the common herbal medicines used for the treatment of gastrointestinal disorders [13–15]. Many *in vivo* and *in vitro* studies have shown the antispasmodic effects of these herbs [15–19]. Another study on rats has proven the ability of TA to reduce the time of food transit through the gastrointestinal (GI) tract [14, 20, 21]. Also, various studies indicate the anti-inflammatory, antioxidant, and analgesic effects of all the three plants used in this study and the monoterpenes in their essential oils [22–24]. Considering the characteristics mentioned for these medicinal herbs and since many of the medicines used to treat IBS are based on the relief of pain and muscle spasm, reduction of inflammation, improvement of gastrointestinal motility, and modification of intestinal flora, the present study is aimed at investigating the effects of a mixture of the essential oil of these herbs on IBS symptoms in a clinical trial.

## 2. Patients and Methods

### 2.1. Study Design

The present study was a randomized double-blind clinical trial with parallel groups and an allocation ratio of 1 : 1 in Iran, from November 2017 to May 2018. The Medical Ethics Committee of Hormozgan University of Medical Sciences approved the study protocol according to the guidelines of the International Conference on Harmonization and the ethical principles originating in the Declaration of Helsinki with number HUMS.REC.1394.012. The trial was registered in the Iranian Registry of Clinical Trials with trial ID number IRCT2016072629026N3. All authors had access to the study data and reviewed and approved the final manuscript.

### 2.2. Study Participation

The statistical population included all patients aged between 15 and 65 years who were visiting the Gastroenterology Clinic of Shahid Mohammadi Hospital of Bandar Abbas. Inclusion criteria were complete knowledge about the study and written consent; being diagnosed with IBS based on the Rome III criteria; IBS Symptom Severity Scale (IBS-SSS) score of ≥175 (moderate to severe IBS); and absence of alarm features such as a history of gastrointestinal bleeding, increasing pain or nocturnal pain that wakes up the patient or prevents sleeping, severe weight loss in the previous six months, family or person history of colorectal cancers or chronic gastrointestinal diseases (such as persistent diarrhea and inflammatory bowel disease), abnormal laboratory finding such as unreasonable anemia, elevated erythrocyte sedimentation rate (ESR), positive celiac serology, and increased fecal calprotectin [4, 25]. Exclusion criteria were the participants' lack of consent to continue their participation in the study; pregnancy or breastfeeding; kidney and liver diseases based on laboratory tests; a history of a severe allergic reaction to medicinal plants; serious illnesses like heart failure and diabetes; previous or current significant psychiatric comorbidity; regular antibiotic usage and the use of hyoscine butylbromide or herbal medicine within the preceding one month; thyroid disease; and chronic consumption of medication that could interfere with intestinal motility, secretion, and sensation. [5, 6, 25, 26].

### 2.3. Randomization, Blinding, and Intervention

At first, the patients were visited by a gastroenterologist, and those who met the inclusion criteria were included in the study. After obtaining informed consent, patients were randomly divided by using random allocation software with an allocation ratio of 1 : 1 into two groups (control and intervention). Also, control and intervention groups received medical regimens A and B, respectively. The randomization and medicine administration were done by someone other than the investigators. The researchers, patients, and the treatment assessor were not aware of the type of medication that each group was taking. Medicines were put in the same cans labeled with the relevant code. The code of medicine given to each patient and their clinical symptoms were recorded on the personal information form by the treatment assessor (a trained medical student). Patients in the control group received three tablets of 10 mg hyoscine butylbromide every day for two weeks. Those in the intervention group were daily treated with two 250 mg softgel capsules containing sunflower oil (28%) as an excipient and pure essential oils of ZM (28.8%), AG (21.6%), and TA (21.6%) for two weeks. Also, for blinding the patients, aromatized sunflower oil softgel capsules twice a day and starch tablets three times a day in the same shape, size, and color as placebo were given to the control and intervention arms, respectively.

### 2.4. Preparation of the Herbal Softgel Capsule

250 mg softgel capsules containing sunflower oil (28%) as an excipient and pure essential oils of ZM (28.8%), AG (21.6%), and TA (21.6%) were produced in Minoo Pharmaceutical Company (Tehran, Iran). The essential oils were purchased from Barij Essence Pharmaceutical Company (Kashan, Iran). These essential oils were prepared by the hydrodistillation method (Clevenger apparatus) from the fruits of TA and aerial parts of AG and ZM.

### 2.5. Determining the Safe Dosage

According to the available resources, the maximum dose of essential oils of the studied plants in humans is equal to 500 mg for TA, 300 mg for AG, and 240 mg for thyme for daily oral consumption [26–29]. Based on our toxicity study before the clinical trial, acute oral toxicity (LD50) was obtained for the mixture of these essential oils at 4250 mg/kg and the intraperitoneal injection of this mixture up to 250 mg/kg/day for three weeks caused no disturbance in liver enzymes and lactate dehydrogenase (Bordbar et al. 2016). Finally, it was decided to treat patients with 360 mg/day of the mixture.

### 2.6. Identification and Separation of Essential Oil Compounds

The filtered essential oils were subjected to gas chromatography-mass spectrometry (GC-MS) analyses. GC-MS was carried out on an HP 5890 GC system coupled to a quadrupole mass detector. Helium was used as a carrier gas in a constant flow mode at 1 ml/min. The temperature of the column in the initial procedure was 70°C, which was gradually increased by 10°C up to 280°C. The instrument has been set to 70°C as the initial temperature for 2 min. Then, the temperature has been raised to 280°C, at an increased rate of 5°C/min to 9 min. Also, the ionization voltage was 70 eV. The separation was achieved with the RTS-volatile column about 30 m long. A quadrupole mass detector was employed to detect compounds when they were vented from the column. The temperature of the detector was 300°C. Using data libraries such as the NIST (National Institute of Standards and Technology) database, the mass spectra obtained through GC-MS were analyzed, and the volatile compounds of the plant samples were identified [30, 31].

### 2.7. Evaluation of Outcomes

The patients were asked to fill out the IBS-SSS at the beginning, at the end, and two weeks after the end of the intervention. Also, seven days before the start and end of the intervention, the participants were asked to record their daily symptoms. Daily symptoms included daily abdominal bloating and abdominal pain (number of days with abdominal pain and bloating), stool frequency (number of stools per day), and daily stool diary. The daily stool diary was used to record stools based on the Bristol Stool Form Scale for IBS subtyping and mean stool consistency on a 7-point scale [32, 33].

Also, they completed the IBS Adequate Relief (IBS-AR) and IBS Global Assessment Improvement (IBS-GAI) questionnaires at the end and two weeks after the end of the study. To investigate the effect of treatment on the quality of life in patients, they were asked to fill out the 36-Item Short-Form Health Survey questionnaire (SF-36).

#### 2.7.1. IBS-AR

This scale consists of a simple yes/no question that is asked at the end of the study: “Have you felt enough recovery in your symptoms over the past seven days?” “Yes” was regarded as a positive response to treatment [34].

#### 2.7.2. IBS-GAI

This tool considers the IBS symptoms thoroughly and presents the results in grades. At the end of the trial, patients are asked the following question: “How do you rate the improvement of your symptoms during the last week compared to what you normally felt before the treatment?” Patients answer the question based on a 7-point scale from “much better” to “much worse.” Options “much better” and “moderately better” were regarded as a response to treatment [35, 36].

#### 2.7.3. IBS-SSS

This 5-item scale measures abdominal pain intensity, abdominal pain frequency, abdominal bloating/distension, satisfaction with the improvement of stool excretion, and the impact of IBS on the quality of life using the visual analogue scale. The overall score of this scale ranges between 0 and 500, and higher scores indicate higher severity of the disease. Also, based on previous studies, the minimal clinically important difference (MCID) from baseline to postintervention is defined as a reduction in the total score of ≥95 points after the treatment [37, 38]. Accordingly, this amount of MCID after the intervention was considered a positive response to treatment.

#### 2.7.4. SF-36

This questionnaire assesses physical and mental health. The total score of this questionnaire is obtained by combining the scores of the eight areas of the questions. In addition to the total score, two scores for the physical and mental sections are calculated based on a specific guideline. Questions are scored on a 5-point Likert scale from excellent (100) to poor (0) [39, 40].

Primary outcomes were the response rates based on the IBS Symptom Severity Scale (IBS-SSS), IBS Adequate Relief (IBS-AR), and IBS Global Assessment Improvement (IBS-GAI) at the end and two weeks after the end of the intervention. Secondary outcomes were the improvement rates in IBS-SSS scores, daily symptoms, and stool consistency, improving the quality of life, safety, and tolerability.

### 2.8. Safety and Compliance

Mild and severe adverse events were documented to evaluate the safety of treatment regimens. If there were severe adverse events, the treatment was discontinued. Laboratory tests (serum alanine aminotransferases, aspartate aminotransferases, alkaline phosphatase, total and direct bilirubin, blood urea nitrogen, creatinine, and random blood sugar) were taken at the beginning and the end of the study to ensure the safety of treatment regimens. Also, if patients had taken more than 80%, 60-80%, and less than 60% of the prescribed medications by the end of the study, acceptance rates were considered full, good, and poor, respectively [41].

### 2.9. Sample Size and Statistical Analyses

Based on a pilot study before this trial, the treatment rate of IBS by this herbal medicine was obtained at 83%. Also, to determine the sample size, the treatment rate with hyoscine [42], confidence level, and power were considered 45%, 0.05, and 90%, respectively. According to this, the sample size was estimated to be 31 in each group. Statistical analysis was performed using SPSS version 17. Independent samples *t*-tests, Mann-Whitney *U* tests, and paired sample *t*-test were used to compare quantitative variables, and the chi-square test was used to compare qualitative variables. A *P* value of less than 0.05 was considered statistically significant. The odds ratio (OR) was calculated using logistic regression and 2-sided 5% type I error. The efficacy analysis was based on the intention-to-treat (ITT) population, including all patients who had at least one observation after the start of treatment, with missing values replaced by the mean imputation method [43].

## 3. Results

### 3.1. Patient Flow and Baseline Characteristics

**Figure 1:**
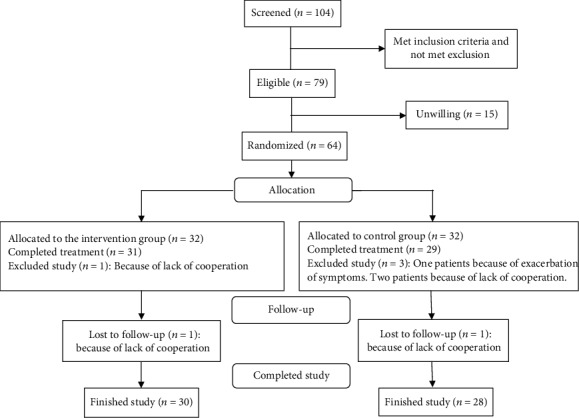
Flowchart illustrating the progress of patients through the study.

Figure 1 shows the patient flowchart, according to the CONSORT Statement advice.

A total of 104 patients with IBS were screened for this trial from November 2017 to May 2018, of whom 79 met the inclusion criteria. However, 15 patients were not willing to participate in the study, and finally, 64 patients (32 patients in the intervention group and 32 patients in the control group) were randomized to receive treatment. Demographic details and IBS-related summary measures are shown in Table 1.

During the first two weeks of the study, in one of the patients from the intervention group (due to lack of cooperation) and three patients from the control group (two cases due to lack of cooperation and one due to worsening of symptoms (constipation and abdominal pain)), no efficacy assessment was performed. These patients were excluded from the efficacy evaluation. Lack of cooperation was defined as not completing the checklist of daily symptoms or not visiting after treatment. Thus, the statistical analysis of efficacy was performed with 60 patients (31 in the intervention group and 29 in the control group). Also, two patients did not return for reassessment after the first assessment on day 14. In these cases, missing values were replaced by the mean imputation method. As a result, 90.62% of patients (30 in the intervention group and 28 in the control group) finished the study. The trial was terminated in May 2018 after reaching its recruitment goal.

### 3.2. Outcomes

**Figure 2:**
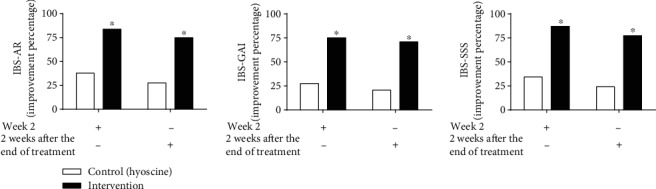
The response rate to treatment based on IBS-AR, IBS-GAI, and IBS-SSS scales. Intention-to-treat population. IBS-AR: IBS Adequate Relief; IBS-GAI: IBS Global Assessment Improvement; IBS-SSS: IBS Symptom Severity Scale. ^∗^*P* < 0.001, intervention vs. control. Control (*n* = 29). Intervention (*n* = 31).

Concerning IBS-AR and IBS-GAI scales, the improvement rate after the end of the treatment and two weeks after the end of the treatment was significantly higher in the intervention group than in the control group (Figure 2). Also, regarding the IBS-SSS scale, based on the MCID ≥ 95 points after the treatment as a response to treatment, the improvement rate after the end of the treatment and two weeks after the end of the treatment was significantly higher in the intervention group than in the control group (Figure 2). Regarding the posttreatment improvement based on IBS-AR and IBS-GAI scales, the improvement chance in the intervention group was 8.5 times and nine times higher than that in the control group (OR 8.5, CI 95% (2.5-28.7), *P* = 0.001 and OR 9, CI 95% (2.7-29), *P* < 0.001, respectively). Also, two weeks after the end of the treatment, the improvement chance based on the IBS-AR and IBS-GAI scales in the intervention group was nine times and 9.3 times higher than that in the control (OR 9, CI 95% (2.7-29), *P* < 0.001 and OR 9.3, CI 95% (2.8-30.7), *P* < 0.001, respectively).

**Figure 3:**
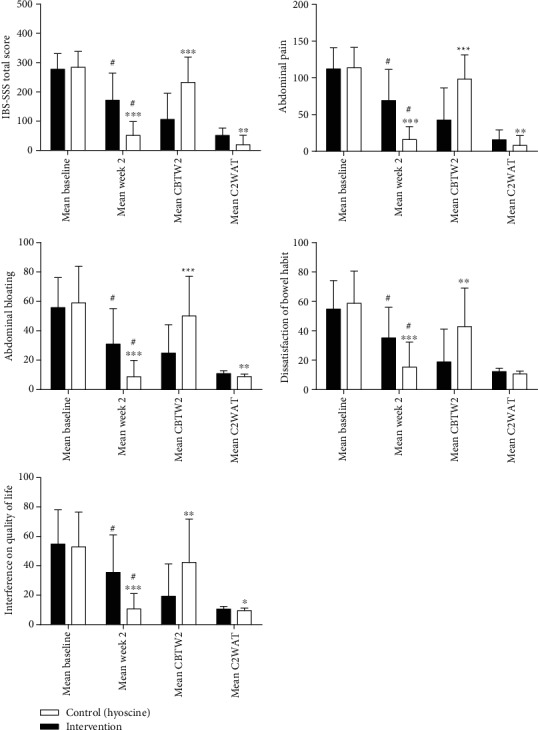
The mean of total and subscale scores of IBS-SSS. Intention-to-treat population. IBS-SSS: IBS Symptom Severity Scale; mean CBTW2: mean change baseline to week 2; mean C2WAT: mean change 2 week after treatment. ^∗^*P* < 0.05, control vs. intervention; ^∗∗^*P* < 0.01, control vs. intervention; ^∗∗∗^*P* < 0.001, control vs. intervention; ^#^*P* < 0.001, week 2 vs. baseline. Control (*n* = 29). Intervention (*n* = 31).

The mean reduction in the IBS-SSS total score and IBS-SSS subscale scores after the trial was significantly higher in the intervention group (Figure 3). Also, two weeks after the end of the trial, the mean increase in the IBS-SSS total score and IBS-SSS subscale scores was lower in the intervention group. These differences were statistically significant, except for the mean increase in dissatisfaction of the bowel habit score (Figure 3).

The mean reduction in the scores of daily abdominal pain and bloating after the intervention was significantly higher in the herbal medicine group (Table 2). Also, the mean reduction in scores of stool frequency and stool consistency in IBS-D patients after the intervention was significantly higher in the intervention group, and the mean increase in scores of stool frequency and stool consistency in IBS-C patients after the treatment was significantly higher in the herbal medicine group (Table 2).

The improvement of the SF-36 total score and subscale scores of mental and physical health in the intervention group was significantly more than that in the control group (Table 3).

Quantization of the active ingredients measured by GC-MS analysis in each of the essential oils is in Table 4.

### 3.3. Safety and Compliance

In terms of minor adverse effects, total adverse events in the intervention and control groups were two (6.24% of subjects) and 10 (18.75% of subjects), respectively (*P* = 0.12) (Table 5). There were no abnormalities in the results of laboratory tests of patients after the treatment. Medicine compliance in intervention and control groups was full in 93.7% and 90.6%, respectively.

## 4. Discussion

This study was aimed at comparing the efficacy of a new herbal compound composed of ZM, AG, and TA essential oil and hyoscine in improving the symptoms of patients with IBS.

Based on IBS-AR, IBS-GAI, and IBS-SSS scales, the posttreatment improvement percentage was 83.9%, 75%, and 87% in the intervention group and 37.9%, 27.5%, and 34.4% in the control group, respectively (*P* < 0.001). Regarding the improvement of IBS symptoms with hyoscine, previous studies also reported a 45% posttreatment improvement with hyoscine 10 mg four times a day for four weeks, which is close to the results of our study [42, 44]. However, according to the results of a meta-analysis, the rate of posttreatment improvement based on IBS-GAI with an *Aloe vera* extract, *Asafoetida*, and peppermint essential oils was 45%, 63%, and 64%, respectively [45]. These values are lower than the results for the herbal medication used in this study. Also, according to the results of this meta-analysis, 13.4% of patients treated with peppermint essential oil reported adverse events. However, in our study, total adverse events in the herbal medicine group were present in 6.24% of the subjects.

Inflammation, stress-induced changes in autonomic nervous and immune systems, overgrowth and alteration of the gut microbiome, and malabsorption are considered the most important causes of sensory-motor disorders of GI in the IBS [4, 46, 47]. However, various studies indicate the anti-inflammatory, antioxidant, analgesic [22–24], antibacterial [13, 14, 48], and antianxiety [49–51] effects of all the three plants used in this study and the monoterpenes in their essential oils. Probably the efficacy of this herbal medicine is related to these properties.

In this trial, the mean reduction in the IBS-SSS total score and IBS-SSS subscale (abdominal pain, abdominal bloating/distension, and impact of IBS on the quality of life) scores after the trial was significantly higher in the intervention group (Figure 2). Based on the results of this study also, the mean scores of stool frequency and stool consistency in IBS-D patients after the trial were significantly lower in the herbal medication arm than in the hyoscine arm. Also, the frequency of daily abdominal pain and bloating after the trial was significantly lower in the intervention group. Regarding diarrhea-predominant IBS symptoms and symptoms associated with gas retention in IBS, such as bloating and abdominal pain, spasmolytic and anticholinergic medications are used to treat these symptoms [4, 7]. Besides, there are reports on the antispasmodic effects of ZM, TA, and AG due to the anticholinergic and voltage-dependent calcium channel-blocking properties [15–19]. However, antispasmodic and anticholinergic medications cannot be prescribed for all types of IBS, particularly in IBS-C cases, due to the probability of worsening of some symptoms [4, 7]. However, in this trial, the herbal medication, in addition to gas retention symptoms and IBS-D symptoms, also improved the mean scores of stool frequency, stool consistency, and dissatisfaction of bowel habit in IBS-C patients after the trial significantly relative to baseline and significantly higher than those in the control group. This is probably due to the presence of monoterpenes such as thymol, carvacrol, (+)-carvone, and limonene with agonistic effects on the transient receptor potential ankyrin 1 (TRPA1) channel [24, 52, 53]. TRPA1 agonists can stimulate the production of 5-hydroxytryptamine (5-HT) and trigger the serotonergic pathway through influencing TRPA1 channels on the surface of the enterochromaffin cells [54–56]. Activation of the serotonergic pathway by TRPA1 agonists, in turn, activates the gastrocolic neural reflex and colonic motility through stimulation of the 5-HT3 receptors at the terminals of the vagus efferent neurons [56–59]. Also, in animal studies, TA reduced the passage of food in the GI tract [14, 21, 60]. Literature has also shown the efficacy of tegaserod as a 5-HT4 agonist in improving constipation, pain, and bloating in constipation-predominant IBS [61, 62]. So, prokinetic properties, along with antispasmodic properties of these plants, have probably eliminated the limitation of the use of antispasmodic and anticholinergic medications in the treatment of all types of IBS.

As a limitation of our study, the duration of treatment was shorter than that of other trials and treatment regimens to treat IBS. So, the adverse events and compliance of this herbal medication in long-term use need further investigation, although the choice of this short course of treatment was due to the high efficacy of this herbal medicine in the short period in pilot studies before this trial. Also, it is considered that IBS is usually a chronic disease, and discontinuing the intervention may lead to the recurrence of the symptoms. Therefore, there is a need for longer follow-up duration. So, due to the above limitations and other limitations such as low sample size and single-center design of our study, a larger multicenter trial with longer duration of treatment and follow-up is needed to confirm the results of this trial.

## 5. Conclusion

According to the results, the herbal medicine investigated in this study significantly improved the symptoms of IBS more than hyoscine. Therefore, it can be considered an appropriate alternative treatment for IBS.

## Figures and Tables

**Table 1:** Baseline characteristics and IBS-related summary measures of randomized patients.

Baseline information	Intervention group (*n* = 32)	Control group (*n* = 32)	*P* value
Mean age (years), mean ± s.d.	32.8 ± 9.7	35.7 ± 9.1	0.23
Females, *n* (%)	19 (59.37%)	20 (62.5%)	0.47
BMI (kg/m^2^), mean ± s.d.	23.1 ± 2.9	23.4 ± 3.2	0.58
IBS disease duration (years), *n* (%)			
<1	9 (28.12)	12 (37.5)	
1-5	15 (46.87)	13 (40.62)	
>5	8 (25)	7 (21.87)	
IBS subtype by predominant bowel habit, *n* (%)			
IBS-D	10 (31.2)	13 (40.6)	
IBS-C	8 (25)	7 (21.8)	
IBS-M/U	14 (43.7)	12 (37.5)	

IBS: irritable bowel syndrome; IBS-SSS: IBS Symptom Severity Scale; IBS-D: diarrhea-predominant IBS; IBS-C: constipation-predominant IBS; IBS-M/U: IBS with mixed symptoms/unsubtyped IBS; s.d.: standard deviation.

**Table 2:** The mean scores of daily symptoms and stool consistency. Intention-to-treat population.

	Group		Mean baseline (±s.d.)	*P*	Mean week 2 (±s.d.)	Mean CBTW2 (±s.d.)
Daily abdominal pain frequency per week	Control (*n* = 29)		4.8 (1)	0.58	3 (1.8)^¶^	1.8 (1.8)
	Intervention (*n* = 31)		4.9 (0.9)		1 (1.2)^∗∗∗^^¶^	3.8 (1.4)^∗∗∗^

Daily abdominal bloating frequency per week	Control (*n* = 29)		4.9 (1.2)	0.61	2.9 (1.7)^¶^	2 (1.7)
	Intervention (*n* = 31)		5 (1)		0.8 (1.1)^∗∗∗^^¶^	4.2 (1.6)^∗∗∗^

Stool frequency per day	IBS-D	Control (*n* = 12)	4 (0.4)	0.47	3.2 (0.4)	0.7 (0.5)
		Intervention (*n* = 11)	4.2 (0.6)		2.2 (0.3)^∗∗∗^	1.9 (0.7)^∗∗^
	IBS-C	Control (*n* = 6)	0.8 (0.4)	0.66	0.9 (0.2)	0.04 (0.2)
		Intervention (*n* = 8)	0.7 (0.3)		1.8 (0.7)^∗∗^	1 (0.5)^∗∗^
	IBS-M/U	Control (*n* = 11)	2.6 (0.5)	0.37	1.9 (0.2)	0.6 (0.5)
		Intervention (*n* = 12)	2.7 (0.6)		2.5 (0.4)^∗∗^	0.1 (0.7)

Stool consistency	IBS-D	Control (*n* = 12)	5.1 (0.56)	0.68	4.6 (0.42)	0.4 (0.3)
		Intervention (*n* = 11)	5.2 (0.6)		4.3 (0.11)^∗∗^	0.8 (0.5)^∗^
	IBS-C	Control (*n* = 6)	3 (0.31)	0.78	3 (0.12)	0.01 (0.3)
		Intervention (*n* = 8)	2.9 (0.30)		3.6 (0.49)^∗∗^	0.6 (0.3)^∗∗^
	IBS-M/U	Control (*n* = 11)	4.1 (0.22)	0.51	3.6 (0.37)	0.4 (0.5)
		Intervention (*n* = 12)	4 (0.37)		4.1 (0.18)^∗^	0.09 (0.3)^∗^

*P*: *P* value; mean CBTW2: mean change baseline to week 2; control: hyoscine; intervention: herbal medicine. ^∗^*P* < 0.05, control vs. intervention; ^∗∗^*P* < 0.01, control vs. intervention; ^∗∗∗^*P* < 0.001, control vs. intervention; ^¶^*P* < 0.001, week 2 vs. baseline.

**Table 3:** The mean score of SF-36, SF-36PH, and SF-36MH. Intention-to-treat population.

	Group	Mean baseline (±s.d.)	*P*	Mean week 2 (±s.d.)	Mean CBTW2 (±s.d.)	Mean week 4 (±s.d.)	Mean C2WAT (±s.d.)
SF-36	Control (*n* = 29)	76.3 (5.5)	0.95	85.8 (5.6)	9.5 (6.1)	79.9 (7)	5.9 (3.7)
	Intervention (*n* = 31)	76.8 (6)		92.3 (4.8)^∗^	15.6 (6.8)^∗^	90.3 (5.2)^∗^	2 (2.9)^∗^

SF-36PH	Control (*n* = 29)	74.5 (6.7)	0.47	84.9 (6.2)^∗^	10.7 (6.4)	78.8 (7.4)	6 (4.1)
	Intervention (*n* = 31)	75.7 (8)		92.1 (5)^∗^	16.4 (8.9)^∗^	90.5 (6)^∗^	1.7 (2)^∗^

SF-36MH	Control (*n* = 29)	76.2 (7)	0.90	86.6 (5.9)	11.1 (6.2)	80.8 (6.8)	4.7 (4.5)
	Intervention (*n* = 31)	76.4 (6.9)		92.4 (5.6)^∗^	16 (7.2)^∗^	91.4 (4.7)^∗^	1.9 (2.5)^∗^

*P*: *P* value; SF-36: 36-Item Short-Form Health Survey; SF-36PH: 36-Item Short-Form Health Survey physical health; SF-36MH: 36-Item Short-Form Health Survey mental health; mean CBTW2: mean change baseline to week 2; mean C2WAT: mean change 2 week after treatment; intervention: herbal medicine; control: hyoscine. ^∗^*P* < 0.001, intervention vs. control.

**Table 4:** Composition of essential oil with retention time and percentage of each compound.

Compounds	EO			RI^∗^
	ZM%	TA%	AG%	
*α*-Pinene	2.42	0.24	1.10	931
*β*-Pinene	—	1.48	0.80	980
Myrcene	1.53	0.46	—	986
*α*-Phellandrene	—	—	15.76	1008
*ρ*-Cymene	8.64	21.67	0.89	1021
Limonene	—	—	16.85	1032
*β*-Phellandrene	—	—	3.32	1037
*γ*-Terpinene	12.27	20.31	—	1055
Linalool	3.52	—	—	1096
Dill ether	—	—	5.29	1190
*trans*-dihydrocarvone	—	—	8.34	1205
Carvacrol methyl ether	1.12	—	—	1240
D-Carvone	—	—	33.18	1247
Thymol	30.34	50.26	—	1292
Carvacrol	28.84	1.34	—	1304
Dill apiol	—	—	6.80	1628

EO: essential oil; ZM: *Zataria multiflora* Boiss; TA: *Trachyspermum ammi* L.; AG: *Anethum graveolens* L.; RI: the retention index of compounds on the HP-5 column.

**Table 5:** Adverse events in the intervention and control groups.

Safety-evaluable patients^∗^	Control (*n* = 32), *N* (%)	Intervention (*n* = 32), *N* (%)
Serious adverse events	0	0
Epigastric pain	0	1 (3.12)
Abdominal pain	2 (6.24)	1 (3.12)
Constipation	2 (6.24)	0
Xerostomia	4 (12.5)	0
Dizziness	2 (6.25)	0
Total number of adverse events	10 (31.25)	2 (6.24)
Total number of subjects experiencing adverse events^∗∗^	6 (18.75)	2 (6.24)

∗ includes all patients who received at least one dose of study medication. ^∗∗^*P* value = 0.12. Control: hyoscine; intervention: herbal medicine.

## Data Availability

The datasets used and/or analyzed during the current study are available from the corresponding author on reasonable request.

## Supplementary Materials

Supplementary Materials CONSORT checklist herbal medicine intervention.
